# Complete mitochondrial genome of giant cricket *Tarbinskiellus portentosus* (Orthoptera: Gryllinae) and phylogenetic analysis

**DOI:** 10.1080/23802359.2022.2030819

**Published:** 2022-02-03

**Authors:** Cheng-Ye Wang, Pan-Li Yang, Zhao He, Long Sun, Min Zhao, Ying Feng

**Affiliations:** a Institute of Highland Forest Science, Chinese Academy of Forestry, Kunming, PR China; b Key Laboratory of Breeding and Utilization of Resource Insects, National Forestry and Grassland Administration, Kunming, PR China; c College of Forestry, Nanjing Forestry University, Nanjing, PR China

**Keywords:** Mitogenome, *Tarbinskiellus portentosus*, edible cricket, phylogeny

## Abstract

*Tarbinskiellus portentosus* (Lichtenstein, 1796) is an agricultural and forestry pest, but people in some places use it as a delicacy. The complete mitogenome of *T. portentosus* (GenBank accession number MZ427921) is 15, 498 bp in size, including 13 protein-coding genes, 22 transfer RNAs, two ribosomal RNAs genes, and a noncoding A + T-rich region. The A + T-rich region is located between *12S rRNA* and *tRNA^Ile^*. The base composition of the whole *T. portentosus* mitogenome is 40.62% for A, 9.87% for G, 32.20% for T, and 17.31% for C, with a high AT content of 72.82%. The phylogeny analysis indicated that *T. portentosus* had a close relationship with *Cardiodactylus muiri*. The present data could contribute to further detailed diversity and phylogeographic analysis for this edible cricket.

*Tarbinskiellus portentosus* (Lichtenstein, 1796), with the common names of short-tail or giant cricket, is an agricultural and forestry pest, but people in some places use it as a delicacy (Soren et al. 2021). *T. portentosus* belongs to Orthoptera: Grylloidea: Gryllidae (Myers et al. 2021). The protein content of the adult *T. portentosus* is 58%, which made it a potential edible insect (Magara et al. 2020). *T. portentosus* belongs to Orthoptera: Gryllidae: Gryllinae, and the reported distribution including China, India, Malaya, Myanmar, Nepal, Pakistan, and Vietnam (Cigliano et al. 2020). Elucidating the sequence and structure of *T. portentosus* mitogenome is important for diversity and phylogeographic analysis of this edible cricket.

The specimen of *T. portentosus* in this work was obtained from Baise, Guangxi, China (N 23°25′, E 106°38′), and deposited in the insect specimen room (contact person: Cheng-Ye Wang, email: cywang11@126.com) of Research Institute of Resource Insects with voucher number RIRI-w-20200730. Sequencing work of the complete mitogenome of *T. portentosus* was performed by Illumina Nextseq500 in Beijing Microread Genetics Co., Ltd., with a total data volume 4 G (150 bp Reads). High-quality reads were assembled from scratch using IDBA-UD and SPAdes (Bankevich et al. 2012; Peng et al. 2012). Protein-coding genes (PCGs) of the *T. portentosus* mitogenome were identified using BLAST search in NCBI, and *tRNA* genes were identified using the tRNAscan-SE search server (Schattner et al. 2005). The final assembled mitogenome was also verified on the MITOS web server (Bernt et al. 2013).

The *T. portentosus* mitogenome is 15,498 bp in size (GenBank accession number MZ427921), including 13 typical invertebrate PCGs, 22 transfer RNA genes, two ribosomal RNA genes, and a noncoding control region (A + T-rich). The A + T content of the whole *T. portentosus* mitogenome is 72.82%, showing an obvious AT mutation bias (Nguyen et al. 2020). The A + T-rich region exhibits the highest A + T content (77.58%) in the *T. portentosus* mitogenome.

All the 13 PCGs, 11 PCGs use standard ‘ATN’ as start codons. *COX1* use ‘TCG’ as start codon, and *ND1* use ‘TTG’ as start codon. As to the stop codon, *COX3* and *ND5* use ‘T’ as stop codons, and the reminding 11 PCGs have the common mitochondrial stop codon ‘TAA’. Among the 22 tRNAs, 19 tRNAs could be folded into the typical cloverleaf secondary structures; while, *tRNA^Ser^ (GCU)* had completely lost the dihydrouridine (DHU) stem; *tRNA^Phe^ (GAA)* and *tRNA^Tyr^ (GUA)* had lost the TφC loop. The *12S rRNA* gene is located between the A + T-rich region and *tRNA^Val^*, while the *16S rRNA* is located between *tRNA^Val^* and *tRNA^Leu^*.

Based on the concatenated PCGs sequences, the maximum-likelihood method was used to construct the phylogenetic relationship between *T. Portentosus* and 18 other Gryllinae species, with two Gryllotalpinae species used as outgroups (Figure 1). The phylogenetic analysis was conducted using MEGA version X (Kumar et al. 2018). Potential substitution saturation of PCGs data set was assessed using DAMBE5 software (Xia 2018), and the outcome (Iss (0.072) < Iss.c (0.784), *p* < .05) indicated that the substitutions between sequences did not reach saturation and the sequences can be used for subsequent phylogenetic analysis. The phylogeny analysis indicated that *T. portentosus* had a close relationship with *Cardiodactylus muiri* (Figure 1), which add new information to the evolutionary lineage research of *T. portentosus* (Tantrawatpan et al. 2011; He et al. 2020; Pradit et al. 2021). This mitogenome data might be also valuable for further phylogeography analyses for this edible cricket.

**Figure 1.**
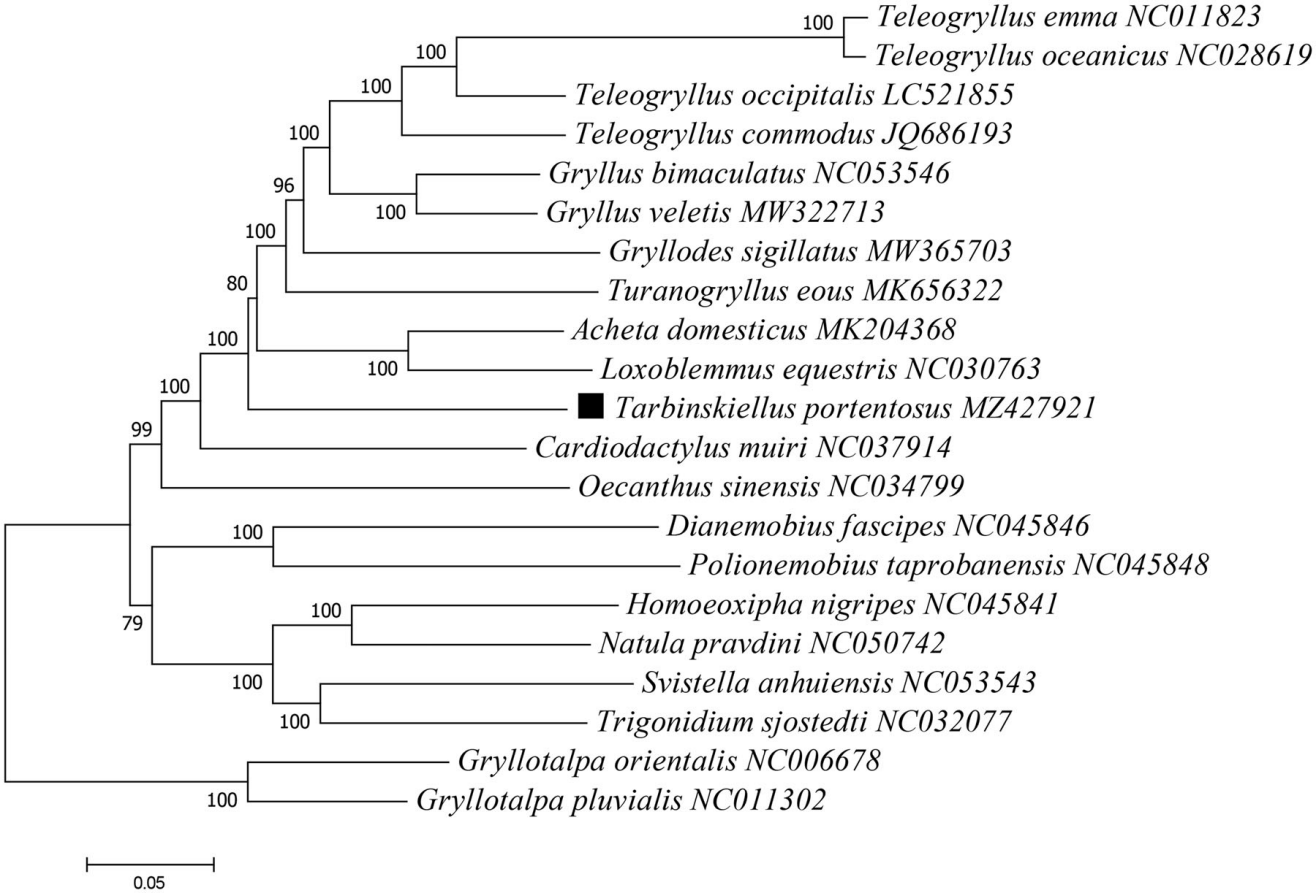
Phylogenetic tree showing the relationship between *T. portentosus* and 18 other Gryllinae species based on maximum-likelihood method performed using 500 bootstrap replicates. Two Gryllotalpinae species (*Gryllotalpa orientalis* and *G. pluvialis*) were used as outgroups. GenBank accession numbers of each sequence were listed in the tree behind their corresponding species names.

## Data Availability

The genome sequence data that support the findings of this study are openly available in GenBank of NCBI at [https://www.ncbi.nlm.nih.gov] under the accession no. MZ427921. The associated BioProject, SRA, and bio-sample numbers are PRJNA741082, SRR14902953, and SAMN19844586, respectively.
